# Chronic Leg Ulcers: Are Tissue Engineering and Biomaterials Science the Solution?

**DOI:** 10.3390/bioengineering8050062

**Published:** 2021-05-10

**Authors:** Christos Kyriakidis, Ferdinand Lali, Karin Vicente Greco, Elena García-Gareta

**Affiliations:** 1 Regenerative Biomaterials Group, The RAFT Institute & The Griffin Institute, Northwick Park and Saint Mark’s Hospital, London HA1 3UJ, UK; c.kyriakidis@btinternet.com; 2 The Griffin Institute, Northwick Park and Saint Mark’s Hospital, London HA1 3UJ, UK; f.lali@griffininstitute.org.uk (F.L.); k.greco@griffininstitute.org.uk (K.V.G.); 3 Division of Surgery and Interventional Science, Royal Free Hospital Campus, University College London, London NW3 2QG, UK; 4 Division of Biomaterials and Tissue Engineering, Royal Free Hospital Campus, Eastman Dental Institute, University College London, London NW3 2QG, UK

**Keywords:** leg ulcers, chronic wounds, diabetic foot ulcers, tissue engineering, dermal scaffolds

## Abstract

Chronic leg ulcers (CLUs) are full thickness wounds that usually occur between the ankle and knee, fail to heal after 3 months of standard treatment, or are not entirely healed at 12 months. CLUs present a considerable burden on patients, subjecting them to severe pain and distress, while healthcare systems suffer immense costs and loss of resources. The poor healing outcome of the standard treatment of CLUs generates an urgent clinical need to find effective solutions for these wounds. Tissue Engineering and Biomaterials Science offer exciting prospects for the treatment of CLUs, using a broad range of skin substitutes or scaffolds, and dressings. In this review, we summarize and discuss the various types of scaffolds used clinically in the treatment of CLUs. Their structure and therapeutic effects are described, and for each scaffold type representative examples are discussed, supported by clinical trials. Silver dressings are also reviewed due to their reported benefits in the healing of leg ulcers, as well as recent studies on new dermal scaffolds, reporting on clinical results where available. We conclude by arguing there is a further need for tissue-engineered products specifically designed and bioengineered to treat these wounds and we propose a series of properties that a biomaterial for CLUs should possess, with the intention of focusing efforts on finding an effective treatment.

## 1. Introduction

Cutaneous wound healing is a complex biological process, reliant on a series of highly regulated and overlapping physiological events including hemostasis, inflammation, proliferation, and tissue remodeling [1]. Disruption at any stage can result in unsuccessful healing and formation of chronic wounds, which are ulcerative skin defects that do not heal in an orderly and timely manner, often failing to heal for more than six months [1]. They frequently remain in the inflammatory stage for an extended period with reduced cell proliferation and deficient response to growth factors [2].

Chronic leg ulcers (CLUs) are full thickness wounds that usually occur between the ankle and knee, fail to heal after 3 months of standard treatment or are not entirely healed at 12 months [3,4]. The frequency of such non-healing ulcers is growing through an increasing ageing population, and factors such as smoking, obesity, and diabetes contribute to the impaired healing. Venous disease is the most common cause of CLUs, accounting for roughly 70% of cases, whilst approximately 20% are caused by arterial disease or mixed arteriovenous disease [5]. Furthermore, peripheral neuropathy accounts for approximately 85% of foot ulcers, which are often complicated by arterial disease [5]. A common complication of diabetes mellitus is the formation of diabetic foot ulcers (DFUs) with an estimated 19–34% of diabetes patients likely to develop DFU in their lifetimes [6].

CLUs present a considerable burden on patients, subjecting them to severe pain and distress, while healthcare systems suffer immense costs and loss of resources. Annual figures by the International Diabetes Federation in 2015 reported that 9.1–26.1 million people will develop DFUs [6]. Approximately 1% of the adult population in developed countries is affected by CLUs and an estimated 3.5 per 1000 individuals affected in the UK, which rises to 20 per 1000 individuals in people over the age of 80 [7,8]. Additionally, the annual national health service (NHS) cost to manage confirmed CLUs such as DFUs was estimated to be between £524.6 and £728.0 million, and for venous leg ulcers (VLUs) between £596.6 and £921.9 million [9,10]. However, these values may be an underestimate since they only consider the confirmed cases, and the NHS also managed patients with lower limb ulcers without a differentiation [9,10]. Moreover, and according to recent published data the worldwide prevalence rate of DFUs was 6.3%. Globally more than 400 million people are suffering from DFUs and the resulting complications and in many cases amputations of extremities, high risk of mortality and morbidity have huge adverse implications on healthcare system and health economics globally [11,12].

In the past, the standard treatment of CLUs involved the use of continuous graduated compression therapy together with dressings [13,14,15]. This treatment method had poor healing outcomes with only 50% of ulcers healing within four months, roughly 20% not healing within two years and about 8% not healing even after five years [16]. Consequently, many patients with unhealed ulcers were admitted in the hospital for treatment, frequently with unsuccessful outcomes leading to lower limb amputation culminating in lifetime disability.

Despite considerable advances in the management of chronic wounds, some of the most promising discoveries still lie ahead. Such advances should lead to the complete anatomical and physiological restoration of the skin. To mention a few, these new approaches and techniques include: (i) cell and gene therapies; (ii) soluble molecules and bioactive factors; and (iii) development of efficient engineered biomaterials at affordable costs and availability.

The fields of Tissue Engineering and Biomaterials Science offer exciting prospects for the treatment of CLUs, using a broad range of skin substitutes or scaffolds. They consist of a group of biomaterials that provide wound cover for tissue repair and regeneration after an injury that extends deeper than the epidermis of the skin [17]. These scaffolds or substitutes vary in their material composition (biological origin, natural or synthetic polymers, ceramics), permanence (temporary or permanent), intended layer of replacement (epidermis, dermis, or both), and the presence or lack of cells [17]. In terms of the material composition, some of the skin substitutes are of biological origin derived from native extracellular matrix (ECM), therefore are formed of native proteins and ligands that provide the physiological microenvironment for the new tissue to grow [18]. Alternatively, biomaterials that are used to develop novel skin scaffolds commonly include polymers (natural or synthetic) and ceramics (bioactive glasses), which all have their advantages and disadvantages [17]. Accordingly, to minimize or eliminate disadvantages, they are frequently combined to form composites that integrate their various distinctive advantages. Specific conditions are required to develop a functional dermal scaffold: biodegradability as new dermis is formed; and survival for a sufficient period so that cells have ample time to infiltrate and deposit a new ECM, without evoking a foreign body reaction. Furthermore, the scaffold should permit cell proliferation and vascularization, withstand tear forces, retain flexibility, and should be easily handled by surgeons and clinical practitioners [19].

Skin substitutes are not the only type of biomaterials that Tissue Engineering and Biomaterials Science can offer for the treatment of CLUs. Wound dressings are a type of skin biomaterials that are temporarily placed above a wound to facilitate the natural wound healing mechanisms and provide an optimal healing environment. They can be made from a vast array of materials and some of them can include components such as antimicrobial agents [20].

In this review, we discuss the various types of scaffolds used clinically in the treatment of CLUs, such as VLUs, arterial leg ulcers (ALUs) and DFUs (Table 1). Their structure and their therapeutic effects will be described. For each type of scaffold, examples will be discussed, supported by clinical trials (Table 1). Moreover, the importance of silver dressings will be reviewed due to their reported benefits in the healing of CLUs. Recent studies on new dermal scaffolds will also be examined, reporting on clinical results available. Finally, we conclude by proposing a series of properties that a biomaterial specifically designed for CLUs should possess, with the intention of focusing efforts on finding an effective treatment.

## 2. Living Skin Substitutes

Living skin substitutes are either natural or synthetic scaffolds seeded with allogenic fibroblasts, and/or keratinocytes. They replace cellular and structural components for wound healing. Examples of living skin substitute products currently used in the treatment of CLUs include Apligraf^®^ (Organogenesis, Inc., Canton, MA, USA), and DermaGraft^®^ (Organogenesis, Inc., Canton, MA, USA) [21].

### 2.1. Apligraf^®^

Apligraf^®^ is a bioengineered bilayered living cellular construct (BLCC). It is a metabolically active skin scaffold providing both a dermal and epidermal layer. The dermal layer is made of a bovine type I collagen matrix seeded with neonatal fibroblasts, on top of which human neonatal epidermal keratinocytes are added forming the epidermal layer [22]. The living cells secrete vital cytokines and growth factors to stimulate differentiation and proliferation [23]. Apligraf^®^ was originally approved by the Food and Drug Administration (FDA) in 1998 for the treatment of VLUs of greater than one-month duration and later in 2000 got approval for the treatment of DFUs of greater than three weeks duration [22].

Recently, Stone et al. hypothesized that treatment with Apligraf might activate responsiveness to cellular signals similar to those that facilitate successful healing of acute wounds, thus changing a non-healing to a healing phenotype. Therefore, they designed a randomized controlled post-marketing clinical trial to investigate the effects of Apligraf on gene expression in chronic VLUs. In their clinical trial, they included 30 participants with VLUs who were treated with the SOC compression therapy for four weeks. Patients with non-healing VLUs, defined as those that did not have 40% reduction in ulcer size with compression therapy over this time period, were enrolled and randomly assigned to receive either ongoing SOC treatment with compression dressings (control group, *n* = 9) or up to five weekly Apligraf applications in addition to the SOC (treatment group, *n* = 15). Biopsies from the wound edge were obtained at baseline (week 0) and week 1 for all ulcers, and they used comprehensive microarray, mRNA, and protein analyses on the samples. They found that ulcers treated with Apligraf displayed three distinct transcriptomic patterns, suggesting that Apligraf induced a shift from a non-healing to a healing tissue response involving modulation of inflammatory and growth factor signaling, keratinocyte activation, and attenuation of Wnt/β-catenin signaling. Overall, they were able to provide new insights into the efficacy of Apligraf in healing chronic VLUs, 15 years after initial pivotal clinical trials suggesting that similar studies can be integrated into clinical trials, providing new foundations upon which existing and novel therapeutic and diagnostic approaches for chronic wound healing can be examined. Their findings provided in vivo evidence in patient VLU biopsies of pathways that can be targeted in the design of new therapies to promote healing of chronic VLUs [24].

Another pilot study evaluated the effectiveness of Apligraf, compared to a living, cryopreserved, human skin allograft (TheraSkin), in conjunction with compression therapy. This study was designed and conducted as a prospective, head-to-head, single site, randomized clinical trial to assess differences in healing rates, adverse outcomes, and treatment costs. They found different but not statistically significant healing rates between the two materials and no adverse outcomes suggesting that TheraSkin which on average costs almost half the price per patient compared to Apligraf may provide equivalent outcomes to Apligraf while reducing costs [25].

### 2.2. DermaGraft^®^

DermaGraft^®^ is a sterile, cryopreserved human fibroblast-derived dermal substitute comprising of fibroblasts, ECM and a bioresorbable polyglactin mesh scaffold. In 2001 DermaGraft^®^ was approved by the FDA for the treatment of DFUs via a randomized, controlled clinical study on 314 patients with a DFU in 35 centers within the US [26,27]. When the human fibroblasts (harvested from neonatal foreskin) proliferate in the spaces within the scaffold, they secrete collagen, ECM proteins, growth factors and cytokines, generating a three-dimensional dermis composed of metabolically active, living cells [28,29,30]. More recent studies on DermaGraft^®^ found that it consists of only human dermal fibroblasts, including their secreted products, but completely lacks other types of cells found in skin such as keratinocytes, lymphocytes, macrophages, and endothelial cells [28].

The effectiveness of DermaGraft^®^ in treating VLUs has also been confirmed and is now approved for marketing in the US. In a prospective, multicenter, randomized controlled study, 186 patients with VLUs were treated with DermaGraft^®^ plus compression therapy, and 180 patients were treated with compression therapy alone (control group) [27]. Both groups were followed up for 12 weeks to determine the proportion of patients with completely healed VLUs. By week 12, 34% of the DermaGraft^®^ group had healed compared with 31% in the control group (*p* = 0.235). In ulcers with less than 12 months’ duration, 52% of patients in the DermaGraft^®^ group healed at 12 weeks compared to 37% of patients in the control group (*p* = 0.029). However, the prevalence of ulcer related adverse events did not notably differ between the two groups [27].

More recently Frykberg et al. conducted a multicenter clinical study to assess the application of MatriStem MicroMatrix (MSMM) and MatriStem Wound Matrix (MSWM) (porcine urinary bladder derived extracellular matrix) compared with DermaGraft^®^ for the management of non DFUs. The study was designed to evaluate the incidence of ulcer closure, rate of ulcer healing, wound characteristics, patient quality of life, cost-effectiveness, and recurrence. They randomized patients whose DFUs decreased in size by ≤30% or increased by ≤50% during the standard of care (SOC) phase and evaluated complete wound closure by eight weeks with weekly device application. A two-week post treatment SOC phase followed the treatment phase for any wounds that did not heal by the end of eight weeks, and wound closure was also evaluated at the end of that period. Ulcer recurrence at six months post-treatment was evaluated in the subjects that showed wound healing by the end of the post-treatment SOC phase. Standard adjunctive therapy, including debridement, saline irrigation, and foot off-loading, was provided to both arms during the four-week screening period, after which eligible subjects were randomized in a 1:1 ratio, to either the MatriStem (MS) or DermaGraft^®^ treatment arm. This study was developed to evaluate the hypothesis that the wound outcomes observed after wound management with MS were non-inferior to those of DermaGraft^®^ after eight weeks. 95 subjects were consented and entered the SOC four-week screening phase of the trial and 56 were randomized into the treatment phase. At the planned interim analysis, there was a significantly lower cost per subject and significant improvement in patient quality of life for the subjects treated with MS compared with those managed with DermaGraft^®^. However, there was not a statistically significant difference found during the analysis of the interim data between the two study groups for rate of wound healing or number of subjects with complete wound closure. The data from this interim analysis show that MSMM and MSWM provide results for healing DFUs that are similar to the results obtained for DermaGraft^®^ at a significant quality of life and economic advantage [31].

In another clinical trial a viable cryopreserved placental membrane (vCPM) and DermaGraft^®^ were investigated for the treatment of Chronic DFUs in order to make parallel comparisons of the clinical and cost effectiveness of two different skin substitutes. This was a multicenter, single-blind study that involved 62 patients (31 in each treatment arm). The results showed no significance difference between the two products in terms of achieving complete wound closure. However, they suggested that the less expensive vCPM may have better outcomes for smaller wounds (≤5 cm^2^) resulting in significant savings in treatment cost [32].

## 3. Acellular Naturally Derived Protein-Based Polymeric Scaffolds

Acellular scaffolds may be derived from animal or human tissues, by decellularization, or they could be synthetic or a composite. For the treatment of chronic wounds, natural protein-based polymers are often used in the development of acellular scaffolds, due to their structural and biochemical similarity to the ECM as well as their mechanical tunability, high biocompatibility, and high-water holding capacity [33]. Examples of acellular naturally derived protein-based polymeric scaffolds used in the treatment of CLUs include Oasis^®^ wound matrix (Healthpoint), Integra^®^ (Integra LifeSciences), and human amniotic membrane.

### 3.1. Oasis^®^ Wound Matrix

The Oasis^®^ wound matrix is a naturally occurring ECM derived from porcine small-intestine submucosa (SIS), processed into an acellular substrate allowing the cells of patients to grow within [34]. The matrix mainly consists of type 1 collagen and other ECM factors—including glycosaminoglycans, proteoglycans, fibronectin, and growth factors such as transforming growth factor-beta (TGFβ) [34,35,36,37]. The Oasis^®^ wound matrix is subjected to meticulous manufacturing processes to render it free of viral contaminants, provide sterility and reduce the risk of animal-to-human disease transmission [34,38,39].

A prospective, randomized, controlled multicenter trial of 120 patients with VLUs was completed to determine the efficacy of Oasis^®^ wound matrix on the healing of VLUs [39]. The patients were randomized to undergo weekly treatment using Oasis^®^ wound matrix plus compression therapy (Oasis^®^ group, *n* = 62) or compression therapy alone (standard care group, *n* = 58), with healing evaluated weekly for 12 weeks. Treatment with Oasis^®^ wound matrix demonstrated improved results, with 55% of the wounds in the Oasis^®^ group healed, compared with 34% in the standard care group (*p* = 0.0196) [39].

More recently, a study evaluated the clinical safety and effectiveness of Oasis^®^ wound matrix as a treatment for full-thickness pressure ulcers in comparison to standard care. In this clinical trial, 130 adults with Stage III or Stage IV pressure ulcers were randomly assigned and received either multiple topical treatments of Oasis^®^ wound matrix plus standard care (Group A: *n* = 67), or standard care alone (Group B: *n* = 63). The wound healing was assessed weekly for a period of up to 12 weeks, with incidence of complete healing and 90% reduction in ulcer area being the primary outcome measures. The analysis showed a better outcome (40%) in Group A compared to Group B (29%) suggesting that addition of the Oasis^®^ wound matrix to standard care alone might be more beneficial in wound size reduction [34].

Furthermore, a randomized controlled trial compared the complete wound healing effect of either cellular (DermaGraft^®^), and noncellular (Oasis) devices relative to SOC in 56 patients with DFUs over a period of 12 weeks of treatment. This exploratory analysis showed that the outcomes of treatment with either DermaGraft^®^ or Oasis matrix are comparable [40].

### 3.2. Integra^®^ Dermal Regeneration Template

Integra^®^ Dermal Regeneration Template (IDRT) layer is an acellular matrix where the dermal replacement is made of collagen and chondroitin-6-sulfate, engineered to have a regulated porosity and degradation rate [41,42]. The temporary epidermal layer consists of silicone for mechanical stability and for protection against bacterial contamination and water loss. The Foot Ulcer New Dermal Replacement Study laid the foundation of IDRT approval for the treatment of partial- and full-thickness neuropathic DFUs that have not healed for longer than six weeks, with no capsule and tendon or bone exposed [41].

Under an Investigational Device Exemption, a multicenter, randomized, controlled, parallel group clinical trial on 307 patients was conducted to assess the safety and efficacy of IDRT for the treatment of chronic DFUs [41]. The patients were randomized into two groups: control treatment group (SOC treatment: 0.9% sodium chloride gel; *n* = 153) and active treatment group (IDRT, *n* = 154) [41]. The treatment lasted at least 16 weeks and the patients were subsequently followed for 12 weeks. The results documented successful DFU closure, which was markedly greater with IDRT treatment (51%) than with the control treatment (32%; *p* = 0.001) at week 16 [41]. In wounds that healed, it took a shorter time for complete DFU closure for IDRT patients (median time = 43 days) than for patients treated with SOC alone (median time = 78 days) [41]. IDRT treatment not only increased the rate of wound closure but also improved the quality of life of the patients with less ulcer related adverse events compared with SOC treatment.

### 3.3. Human Amniotic Membrane

The human amniotic membrane (HAM) is composed of both amnion and chorion membranes and is found in the inner layer of the placenta [43,44]. The amniotic membrane is avascular, and all nutrients are supplied by diffusion from the amniotic fluid or the underlying decidua. The HAM has five layers, namely epithelium, basement membrane, compact layer, fibroblast layer, and spongy layer [43,44]. The HAM contains numerous growth factors, cytokines, and signaling molecules that are necessary for fetal development and gestation. Importantly, these molecules are also critical in tissue regeneration and wound healing [43,44]. HAM is nonimmunogenic and reduces inflammation and pain while also providing a matrix for cell deposition [43]. The use of HAM products has increased exponentially in the clinical setting, including the treatment of CLUs [43]. For its therapeutic use, HAM needs to be decellularized, with special care taken on preserving the natural ECM of which collagen (various types) is the main component [43,45]. Other proteinic constituents of the HAM are laminin, nidogen, fibronectin, fibulin, fibrillin, perlecan, and agrin, which are all common components of the human ECM [43].

The safety and efficacy of HAM for treating VLUs was investigated in a randomized, controlled trial where HAM plus SOC were compared with SOC alone [46]. The study enrolled 84 patients: 63% were in the HAM plus SOC group and 37% received SOC alone. After four weeks, 62% of patients in the HAM plus SOC group and 32% in the SOC alone group showed >40% wound closure. Furthermore, the HAM plus SOC group had a mean size reduction of 48.1% compared with 19.0% in the SOC alone group. A follow-up study retrospectively examined whether the results at 4 weeks correlated with rates of complete wound healing at 24 weeks. Among patients, 45.4% had wounds that reduced in size >40%, and 55% had a reduction of <40% during the initial study. Of the group with >40% healing at week 4, 80% achieved complete healing in a mean of 46 days, whereas 33.3% of the <40% healing group achieved complete healing in a mean of 103.6 days. This follow-up study thus demonstrated a true correlation of healing between the 4-week and 24-week trials in 73% of patients [47].

An evaluation of the use of HAM on DFUs was done in a prospective, open-label, randomized, parallel group trial that was carried out at eight clinical sites in the USA [48]. Eligible subjects were randomized (1:1) to receive either SOC alone (*n* = 14) or HAM plus SOC (*n* = 15) until wound closure or six weeks, whichever happened first. The endpoint was the proportion of subjects with complete wound closure. In the study, wound closure was defined as complete re-epithelialization without drainage or need for dressings. Results from the trial showed that 35% of the subjects in the HAM plus SOC group achieved complete wound closure at or before week 6, compared with 0% of the SOC alone group (intent-to-treat population, *p* = 0.017). Moreover, 45.5% of the subjects in the HAM plus SOC group achieved complete wound closure, compared with 0% of SOC-alone subjects (*p* = 0.0083). Finally, no treatment-related adverse events were reported. Although the study demonstrated a statistically significant advantage of using HAM in combination with SOC, the authors acknowledged the small sample size and suggested that additional prospective studies were needed [48].

## 4. Acellular Naturally Derived Polysaccharide-Based Polymeric Scaffolds

These scaffolds offer substantial advantages over other scaffolds, including prolonged shelf-life, cost efficacy, and minimal risk of rejection [21,49]. They are designed using polysaccharides which are flexible polymers that mimic the elasticity and porosity of the dermis. Specifically, the functional groups of polysaccharides can be easily altered to create bioactive wound healing biomaterials, which encourage tissue regeneration [50]. In addition, polysaccharides are biocompatible, non-immunogenic, and anti-microbial, thereby promoting more efficient wound healing [50]. Examples of polysaccharide-based scaffolds currently used in the treatment of CLUs include Talymed^®^ (Marine Polymer Technologies) and Hyalomatrix^®^ (Anika Therapeutics).

### 4.1. Talymed^®^

Talymed^®^ is a thin biodegradable and bioactive scaffold consisting of shortened poly-N-acetyl glucosamine (pGlcNAc) fibers acquired from diatom algae [51]. The pGlcNAc fibers are well integrated into the wound bed and cause upregulation of the integrin depended Ets1 transcription factor, which effectively controls cell migration, proliferation, and survival [52]. Furthermore, the shortened pGlcNAc fibers are essential to wound healing as they stimulate endothelial cells and the secretion of cytokines and necessary growth factors, such as interleukin-1 (IL-1) and vascular endothelial growth factor (VEGF) [52]. Recent clinical trials have shown that Talymed^®^ can be also very effective in bone tissue regeneration [53,54].

A randomized, investigator-blinded, parallel group, controlled study on 82 patients with VLUs, was done to evaluate the safety and efficacy of Talymed^®^ in the treatment of VLUs over 20 weeks [55]. The patients were randomized to treatment with standard care alone (*n* = 20), or standard care plus Talymed^®^ applied as follows: (1) only once (*n* = 20); (2) every other week (*n* = 22); (3) every three weeks (*n* = 20). At week 20, 45%, 86.4%, and 65% of VLUs were completely healed for patients in groups 1–3 respectively, compared with 45% for patients receiving standard care alone [55].

### 4.2. Hyalomatrix^®^

Hyaluronic acid (HA) is a non-sulphated glycosaminoglycan (GAG), distributed throughout the ECM, composed of alternating disaccharide units of D-glucuronic acid and N-acetyl-D-glucosamine [56]. HA is known to be a co-regulator for gene expression, proliferation, motility, adhesion, signaling, and morphogenesis [57]. During tissue injury and wound healing, HA synthesis increases and governs many aspects of tissue regeneration, including modulating inflammation, stimulating cell migration, and promoting angiogenesis [58,59,60]. The functional groups of HA are amendable and can be tailored for wound healing applications. Hyalomatrix^®^ is a sterile, flexible wound device comprising of two layers: a non-woven wound contact pad containing fibers of esterified HA and an outer semi-transparent silicone membrane that behaves as a barricade to inhibit vapor loss and diminish bacterial colonization [61]. Hyalomatrix^®^ operates as a regenerative matrix, facilitating accelerated infiltration of fibroblasts and endothelial cells and regulates deposition of the ECM [62,63].

A multicenter, prospective, observational study known as the FAST study, was conducted to evaluate the efficacy of Hyalomatrix^®^ in the treatment of chronic wounds of different etiologies [64]. The study involved the treatment of 262 patients with chronic wounds using Hyalomatrix^®^, with the endpoint being the reduction in threshold area (≥10%) of the ulcer. Out of the 262 ulcers, 121 were vascular ulcers (of which 50% were VLUs, 15% ALUs, and 35% arterial/venous), 66 DFUs (of which 56% were neuroischemic, 27% ischemic, 17% neuropathic), and the rest of different etiology. The outcome of the study was the attainment of re-epithelization (≥10%) in 83% of treated ulcers within 16 days. Therefore, it was proposed that Hyalomatrix^®^ is a safe and effective treatment for CLUs of various etiologies [64].

## 5. Silver-Containing Dressings

Silver-containing dressings are considered extremely important for managing wound infection, since silver is a wide-ranging antibiotic with antiseptic, anti-inflammatory, and healing properties [65]. Back in 2008, a study showed that bacteria isolated from DFUs had a low prevalence of silver-resistant genes suggesting sensitivity to silver. These bacteria are eliminated when exposed to silver-containing wound dressings [66].

A broad range of silver-containing dressings for wound care are nowadays available on the market, such as Urgotul^®^ Silver (Urgo) and AQUACEL^®^ Ag (ConvaTec) [67]. Urgotul^®^ Silver is a sterile, non-adhesive, and non-occlusive dressing, composed of a polyester mesh impregnated with hydrocolloid, petroleum jelly, and silver particles [67]. Urgotul^®^ is a separate version of the dressing devoid of silver. A multi-center, open label randomized controlled trial of 102 patients with VLUs was conducted to evaluate the capacity of Urgotul^®^ Silver to promote healing of VLUs displaying inflammatory signs suggesting critical bacterial colonization [68]. The patients were separated into two groups: treatment and control groups, with treatment lasting eight weeks. Patients in the treatment group (*n* = 51) received Urgotul^®^ Silver for the first four weeks followed by Urgotul^®^ for the following four weeks and the control group (*n* = 48) received only Urgotul^®^ for eight weeks. After eight weeks, the wound area was reduced by 47.9% and 5.6% in treatment and control groups, respectively (*p* = 0.036). Accordingly, the trial demonstrated that treatment with Urgotul^®^ Silver for four weeks results in an increased wound closure rate of critically colonized VLUs [68].

AQUACEL^®^ Ag is a hydrofiber wound dressing incorporating sodium carboxymethylcellulose fibers with ionic silver [69]. The dressing retains moisture, forming a gel upon contact with wound fluid and has antimicrobial properties attributed to the ionic silver. Fibrin gathers at the boundary between the dressing and the wound surface and behaves as an adhesive, holding the dressing in place [69]. A single center, open-label study was conducted on 30 patients with chronic wounds (4 DFUs, 13 VLUs, 4 pressure ulcers, and 9 miscellaneous wounds) to gauge the clinical recovery of these wounds during a four-week application of AQUACEL^®^ Ag [70]. After four weeks, 70% of wounds treated with AQUACEL^®^ Ag decreased in size and there was an increased quality and abundance of healthy granulation tissue [70].

## 6. Current Research

Despite the availability of various clinically used dermal scaffolds, none were explicitly designed for the treatment of chronic non-healing leg ulcers and the effectiveness in treating such ulcers was in most cases moderate. The materials discussed so far in this review were bioengineered for healing of acute wounds that progress through the normal stages of hemostasis, inflammation, proliferation, and tissue remodeling. However, chronic wounds including CLUs do not normally progress through these stages, hence becoming chronic. It has been shown that the chronic wound microenvironment differs from the one found in acute wounds [71]. Some of these differences include high levels of proteases, senescent cells, or reduced levels of growth factors seen in chronic wounds [71]. These differences should be taken into consideration when designing a scaffold for CLUs. For example, the increased level of proteases will affect protein-based scaffolds degradation—i.e., it will accelerate it—thereby degrading the scaffold before a new dermis is formed. Therefore, the bioengineering of a slowly degradable protein-based scaffold for CLUs would be advantageous. Furthermore, successful wound healing depends upon the reestablishment of stable epidermis as a minimum precondition. Stability of the epidermis depends on regeneration of the basement membrane and vascularization to anchor the outer skin to the body [72]. Various skin replacements satisfy many conditions of wound closure; however, they do not recapitulate the multilayered pattern of the skin and adnexa.

Therefore, there is a further need for the development of novel dermal scaffolds specifically designed to target and treat these wounds, with some researchers currently working on creating such scaffolds, which can be combined with stem cells, therapeutic compounds, antibiotics, or exosomes.

### 6.1. Platelet-Rich Plasma

Platelet-rich plasma (PRP) is an autologous concentration of human platelets in a small volume of plasma, consisting of the seven principal protein growth factors including the three isomers of platelet-derived growth factor (PDGFαα, PDGFββ, PDGFαβ), TGFβ1 and TGFβ2, VEGF, and epithelial growth factor (EGF) [73]. These protein growth factors are secreted by platelets to stimulate tissue regeneration and have a valuable therapeutic effect on wound healing [73,74]. Combining PRP and HA in a bio-functionalized scaffold offers several benefits over conventional dressings, including accelerated healing, reduced costs to healthcare systems and a reduction in patient pain [74,75,76].

Burgos-Alonso et al. investigated the efficacy and safety of autologous PRP in comparison with SOC for the treatment of leg ulcers in patients with chronic venous insufficiency, in a primary health-care setting. This was a Phase I–II, open-label, parallel-group, multicenter, randomized pilot study evaluating reduction of ulcer area at five nine weeks after treatment. They have also evaluated the Chronic Venous Insufficiency Quality of Life Questionnaire score, and the cost of treatment for up to nine weeks. Eight patients were recruited and a total of 12 ulcers were treated with either autologous PRP or SOC. They found an increased quality of life in the patients treated, and a reduction in the time of wound care (once a week vs. three times a week in the SOC group) [77].

Moneib et al., compared the clinical efficacy of PRP in the management of chronic venous leg ulcers vs. conventional treatment. In total, 40 patients with chronic venous leg ulcers were included in the study. Twenty patients were treated with autologous PRP weekly for six weeks (Group A), and 20 patients were treated with conventional treatment (compression and dressing) for six weeks (Group B). They showed a highly significant improvement in the ulcer size post-PRP therapy compared to conventional therapy. The mean change in the area of the ulcer post-PRP and conventional therapy was 4.92 ± 11.94 cm and 0.13 ± 0.27 cm, respectively, while the mean percentage improvement in the area of the ulcer post-PRP and conventional therapy was 67.6% ± 36.6% and 13.67% ± 28.06%, respectively. Their data suggested that PRP is a safe nonsurgical procedure for treating chronic venous leg ulcers [78].

In another randomized controlled, open-labeled clinical trial carried out between 2014 and 2018 an eight-week study protocol was chosen or until 100% wound re-epithelialization was observed. A total of 69 patients (35 in the autologous PRP group and 34 in the control group) were included in the study. Wound size reduction, granulation tissue formation, microbiological wound bed changes and safety were evaluated. The autologous PRP group showed superiority over conventional treatment in wound bed coverage with granulation. No severe adverse events were noted during the study. Both treatment methods were considered equally safe suggesting that topical application of autologous PRP gel could be beneficial in wound size reduction and granulation tissue formation. However, treatment was also associated with more frequent microbiological wound contamination [79].

A recent experimental study involved the in vitro and in vivo assessment of a bio-functionalized scaffold composed of PRP and HA, in patients with chronic diabetic and vascular ulcers [74]. The results of patients receiving the combined PRP and HA treatment (*n* = 182) were compared to a control group (*n* = 182) receiving traditional dressings (HA alone). Within 80 days, it was established that patients receiving the combined treatment had 98.4% ± 1.3% re-epithelialization as compared to 87.8% ± 4.1% in the control group (*p* < 0.05). No local recurrence was observed during the follow-up period. The combination treatment showed stronger regenerative potential in terms of epidermal proliferation and dermal renewal as well as an improvement in the healing process in the chronic ulcers compared with HA alone. Overall, the study has shown that combined treatment with PRP and HA could optimize granulation formation and tissue regeneration, provide a more rapid wound closure, an excellent aesthetic improvement, and help preventing infection [74].

### 6.2. Stem Cells

Human mesenchymal stem cells (MSCs) are ideal for tissue regeneration, due to their various exceptional properties, including self-renewal, multilineage differentiation, and immunomodulation [80,81]. Additionally, MSCs occur in almost all tissues including bone marrow, adipose, and synovium [81,82]. Recently, a randomized clinical trial evaluated the effect of a biological scaffold, seeded with human umbilical cord Wharton’s jelly mesenchymal stem cells (WJSCs), in the healing of chronic skin ulcers [83]. Human Wharton’s jelly MSCs (hWJSCs) have numerous advantages over other sources of MSCs such as easy availability, non-invasive isolation, higher growth rate, and lower immunogenicity [83]. Moreover, hWJSCs secrete growth factors involved in wound healing—including VEGF, EGF, PDGF, and hepatocyte growth factor (HGF)—and can differentiate into fibroblast, epithelial, and endothelial cells. The biological scaffold used was of acellular human amniotic membrane, which contains ECM components such as collagen, elastin and fibronectin, known to advance the adhesion, growth, proliferation, and migration of differentiated stem cells in wounds [83,84]. In the study, five patients with chronic DFUs were treated with the scaffold seeded with WJSCs for 9 days, every 3 days with a follow-up of 1 month. Results in treated patients, displayed a noteworthy decrease in wound healing time and wound size, with a mean percent healing of 83.43%, and 96.70% after 6 and 9 days, respectively (*p* < 0.002) [83].

Otero et al. evaluated the feasibility, safety, and initial clinical outcome of autologous bone marrow-derived cells (BMDC) therapy associated with standard treatment in patients with VLUs. They conducted an open-label, single-arm, prospective pilot clinical trial in four patients with six chronic VLUs. Bone marrow was harvested, processed, and then administered by multiple injections into the ulcers. All patients received standard treatment and non-healing characteristics of the VLUs were confirmed at study entry. Both ulcer size and wound pain were significantly reduced 12 months after BMDC treatment, and a long-term follow-up showed that treatment was safe and well tolerated. Despite the low number of patients studied, these data have shown that autologous BMDC treatment could be a useful, feasible, and safe procedure to enhance ulcer healing [85].

Adipose tissue (AT) is a safe and promising tool to treat non-healing venous leg ulcers (VLUs). Zollino et al. conducted a phase II randomized clinical trial for the treatment of recalcitrant chronic leg ulcers using centrifuged AT containing progenitor cells. From an initial cohort of 38 patients, 16 patients affected by non-healing VLUs were randomly allocated to the experimental arm (treatment with AT) and control arm (no treatment). Each group had five men and three women. The primary outcome measures were healing time and safety of the cell treatment. Secondary outcomes were pain evaluated by numeric rating scale (NRS), complete wound healing at 24 weeks by Margolis Index and wound-healing process expressed in square centimeters per week. The various immunophenotypic and functional characteristics of AT-derived stem cells were correlated with the clinical outcomes. The healing time was significantly faster in the AT treated patients (17.5 ± 7.0 weeks) versus 24.5 ± 4.9 weeks recorded in the control arm. No major adverse events were recorded [86].

### 6.3. Hydrogels

Hydrogels and flowable gels are being investigated by numerous research groups as potential materials to treat CLUs. Hydrogels can be natural, synthetic, or hybrid and comprise a three-dimensional network of cross-linked polymers with a hydrophilic structure, which enables them to hold large amounts of water [87,88]. In clinical practice, hydrogels preserve a moist wound environment by supplying water molecules to dehydrated tissue stimulating faster wound healing [88].

A recent study involved the utilization of an engineered hydrogel, known as FHE@exosomes (FHE@exo) hydrogel, for promoting chronic diabetic wound healing and complete skin regeneration [89]. FHE@exo is an injectable, self-healing, and antibacterial polypeptide (poly-ɛ-L-lysine, oxidative HA and Pluronic F127) based hydrogel containing stimuli-responsive adipose-derived mesenchymal stem cells exosomes (AMSCs-exo) [89]. In vivo results demonstrated that the FHE@exo hydrogel notably strengthened the healing of diabetic full-thickness wounds, through amplified wound closure rates, rapid angiogenesis, re-epithelization, and collagen deposition within the wound site [89]. Another study assessed the safety and efficiency of a novel recombinant human type I collagen (rhCollagen) based flowable wound matrix, in patients with CLUs [90]. A single-arm, open-label, multicenter trial was conducted on 20 patients with CLUs, undergoing rhCollagen flowable gel treatment. Wounds were photographed and preliminary surgical debridement was performed prior to rhCollagen application. Patients received a single application of rhCollagen to the wound bed, followed by weekly assessments of the wound—including wound size, granulation, and epithelialization—and application of polyurethane dressing. After four weeks, there was 94% median wound area reduction, with fifteen ulcers showing ≥70% wound closure, nine of which attained complete closure. However, one of the study limitations was that no control group was included to compare with wound healing or with a more traditional product. The authors decided to first assess a short-term applicability and safety and to assess comparative outcomes and long-term follow up in the next trial [90].

## 7. Conclusions

**Figure 1:**
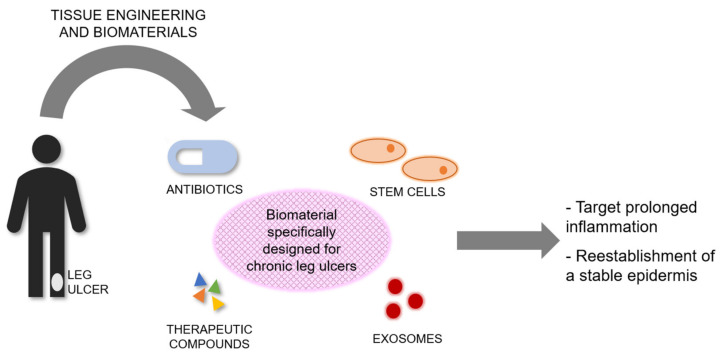
Conceptual scheme highlighting the need for tissue-engineered products specifically designed and bioengineered to treat CLUs. Biomaterials could be combined with antibiotics, therapeutic compounds, stem cells, or exosomes, or a combination of them. Tissue-engineered products for CLUs should target the prolonged inflammation associated with these wounds, followed by the reestablishment of a stable epidermis.

The current standard treatment of CLUs has poor healing outcomes, presenting a considerable burden on patients while healthcare systems suffer immense costs and loss of resources. Tissue Engineering and Biomaterials Science offer exciting prospects for the treatment of these wounds using a broad range of skin substitutes or scaffolds, and wound dressings. In this review we discussed various clinically available and used dermal scaffolds and dressings. However, none were explicitly designed for the treatment of CLUs, and their effectiveness was in most cases limited, indicating a further need for the development of novel biomaterials specifically designed to target and treat CLUs. Although progress is being made, effort is still needed towards the design and development of tissue-engineered products to treat CLUs. Such products should target the prolonged inflammation associated with these wounds, usually due to persistent infection, followed by the reestablishment of stable epidermis (Figure 1). Its stability will depend on regeneration of the basement membrane and vascularization to anchor the outer skin to the body.

## Figures and Tables

**Table 1:** Summary of the types of biomaterials (both skin substitutes or scaffolds, and dressings) used clinically in the treatment of chronic leg ulcers and discussed in detail in this review. The table also provides a description and properties of these scaffolds along with examples.

Type	Description and Properties	Examples
Living Skin Substitutes	-Either natural or synthetic scaffolds seeded with allogenic fibroblasts, and/or keratinocytes. -Replace cellular and structural components necessary for wound healing.	-Apligraf^®^ -DermaGraft^®^
Acellular Naturally Derived Protein-Based Polymeric Scaffolds	-May be derived from animal or human tissues, by decellularization, or could be synthetic or a composite. -Structural and biochemical similarity to the ECM. -Bioactivity, high biocompatibility, mechanical tunability, and high-water holding capacity.	-Oasis^®^ Wound Matrix -Integra^®^ -Human AmnioticMembrane
Acellular Naturally Derived Polysaccharide-Based Polymeric Scaffolds	-Polysaccharides are flexible polymers that mimic the elasticity and porosity of the dermis. -Bioactivity, biocompatibility, non-immunogenicity, and anti-microbial properties.	-Talymed^®^ -Hyalomatrix^®^
Silver-Containing Dressings	-Silver is a wide-ranging antibiotic with antiseptic, anti-inflammatory, and healing properties.	-Urgotul^®^ Silver -AQUACEL^®^ Ag

## Data Availability

Not applicable.
